# The Healing Effect of Adipose-Derived Mesenchymal Stem Cells in Full-thickness Femoral Articular Cartilage Defects of Rabbit

**Published:** 2015-11-01

**Authors:** D. Mehrabani, M. Babazadeh, N. Tanideh, S. Zare, S. Hoseinzadeh, S. Torabinejad, O. Koohi-Hosseinabadi

**Affiliations:** 1 Stem Cell and Transgenic Technology Research Center, Shiraz University of Medical Sciences, Shiraz, Iran; 2 School of Veterinary Medicine, Shiraz University, Shiraz, Iran; 3 Pathology Department, Shiraz University of Medical Sciences, Shiraz, Iran; 4 *Laboratory Animals Center, Shiraz University of Medical Sciences, Shiraz, Iran*

**Keywords:** Transplantation, Mesenchymal stromal cells, Repair, Cartilage, articular, Articular, Rabbits

## Abstract

**Background:**

Articular cartilage defect can lead to degradation of subchondral bone and osteoarthritis (OA).

**Objective:**

To determine the healing effect of transplantation of adipose-derived mesenchymal stem cells (Ad-MSCs) in full-thickness femoral articular cartilage defects in rabbit.

**Methods:**

12 rabbits were equally divided into cell-treated and control groups. In cell-treated group, 2×10^6 ^cells of third passage suspended in 1 mL of DMEM was injected into articular defect. The control group just received 1 mL of DMEM. Dulbecco’s modified Eagles medium (DMEM) supplemented with 10% fetal bovine serum (FBS), 1% penicillin and streptomycin and 2 mM L-glutamine were used for cell culture. To induce cartilage defect, 4 mm articular cartilage full-thickness defect was created in the knee. For histological evaluation in each group (H&E, safranin-O and toluidine blue), 3 rabbits were sacrificed 4 weeks and 3 animals, 8 weeks after cell transplantation.

**Results:**

In cell therapy group post-transplantation, no abnormal gross findings were noticed. Neo-formed tissues in cell-treated groups were translucent with a smooth and intact surface and less irregularity. In cell-treated group after 8 weeks post-transplantation, the overall healing score of experimental knees were superior when compared to other groups.

**Conclusion:**

We showed that Ad-MSCs, as an available and non-invasive produced source of cells, could be safely administered in knee osteochondral defects.

## INTRODUCTION

esions in articular cartilage can result in potentially crippling symptoms such as swelling, pain and decreased mobility and if left untreated, it can lead to osteoarthritis (OA) [1]. So the ultimate goal in OA therapy is to restore the knee function by regeneration of hyaline cartilage in the defect [2]. Various procedures have been described for the treatment of cartilage injuries [3] including microfracture as a minimally invasive and relatively simple treatment measure to perform [4]. One of the main drawbacks in repair of tissue is formation of fibrocartilage rather than hyaline cartilage with poor results in old patients [5] that may be due to insufficient numbers of cells liberated from the subchondral marrow to ensure a durable repair [2] even some authors reported good results but deterioration was visible in function over time [6]. 

More therapeutic alternatives for full thickness cartilage defects were previously described such as resurfacing procedures with perichondrium, periosteum, and osteochondral bone plugs/allografts [2, 7]. Cell-based therapy approaches such as autologous chondrocyte implantation (ACI) emerged as potential treatment option in focal cartilage lesions/injuries [2, 8]. 

However, the procedure has limitations, which include the sacrifice of undamaged cartilage within the same joint and the lack of availability of cell numbers especially in elderly patients. In addition, hyaline cartilage is not always seen in the repair tissue after ACI [2]. 

In treatment of severe cartilage injuries or chondral defects, osteochondral allograft transplantation has been used with good outcomes in clinical practices. However, allograft chondrocyte death due to apoptosis may happen during storage or as a result of implantation stress. Chondrocytes can undergo apoptosis in allo-transplantation. This apoptosis involves the caspase-3 cascade and indicates that chondrocytes may induce acute rejection [9].

Nowadays, most treatments focus on use of cultured cells of different sources to obtain sufficient quantities necessary for tissue regeneration [10]. Mesenchymal stem cells (MSCs) have gained popularity in regeneration of cartilage tissue due to various reasons including their ability to differentiate into connective tissue such as hyaline cartilage and their easy availability isolation from different tissues, *eg*, bone marrow, adipose tissue, cord blood, *etc*. In contrast to articular chondrocytes, the expansion of MSCs does not have higher risk for replicative aging or unlimited growth of these cells making them a candidate for cell-based therapies and an attractive option in regenerative tissue repair [11]. MSCs can be delivered into the knee joint via two different approaches including implantation of the cells directly or via a suitable matrix or scaffold seeded with chondro-progenitor cells and signaling substances and allowing the differentiation process to happen *in vivo*. The alternative would be *in vitro* differentiation of stem cells and implantation of a mature construct [2, 12].

Adipose tissue is an attractive source of stem cells used in regenerative therapies due to high frequency of adipose-derived MSCs (Ad-MSCs) with multilineage differentiation capacity and its abundance [13]. They reside in a supportive stromal vascular fraction (SVF) and are easily isolated [14]. A clinically relevant number of Ad-MSCs can be used in the treatment of osteochondral defects [15] denoting to their ability within the SVF to attach to a scaffold material in sufficient quantities in a short time, and the capacity to differentiate into the osteogenic and chondrogenic lineage [15]. 

Partial-thickness defects evolving in mature articular cartilage do not heal spontaneously and tissue engineering has long been investigated in repair of articular cartilage defects. There are limited treatment choices for cartilage defects in clinical practice due to absence of suitable biomaterials [8]. The current study was designed to evaluate the effect of transplantation of Ad-MSCs in healing of full-thickness femoral articular cartilage defects in experimental rabbit.

## MATERIALS AND METHODS

Twelve mature male Dutch white rabbits aged between 5 and 6 months with a mean±SD weight of 2.20±0.2 kg were provided from the Laboratory Animal Center of Shiraz University of Medical Sciences, Shiraz, Iran. There were six rabbits in the cell-therapy experiment after induction of articular cartilage full-thickness defects and six were allocated to control group. The animals were kept in standard cages, one per cage, in a controlled temperature (20±2 °C) and humidity (55%±5%) with a 12/12-h light/dark cycle. They were allowed to move freely. Standard laboratory chow and tap water were available *ad libitum*. The rabbits were housed, treated and euthanized in compliance with the recommendations of the Animal Care Committee of Iran Veterinary Organization. 

To induce articular cartilage full-thickness defect, 44 mg/kg ketamine (Alfasan, Woerden-Netherlands) and 10 mg/kg xylazine (Rompun, Bayer AG, Leverkusen) were intramuscularly administered under anesthesia while the left leg was shaved and disinfected. Under aseptic conditions on medial para-patellar area of the left knee, a 2-cm incision was made. The patella was displaced laterally to reach the articular capsule. On the femoral articular cartilage, a 4-mm articular cartilage full-thickness defect was created by a trephine while leaving the subchondral bone intact. A 6×6 mm flap was removed from the fascia overlying the quadriceps muscle and was sutured to the peripheral rim of the artificial defect using 6-0 polydioxanone (SUPA, Iran). The skin was closed using 2-0 silk surgical suture (SUPA, Iran). After operation, standard antibiotic (Penicillin, Zakaria laboratory Tabriz, Iran), and analgesic (Flunixin, Razak laboratories, Tehran-Iran) were administered for all rabbits; they were allowed to resume normal cage activity for 12 weeks. 

Under anesthesia induced by intramuscular administration of 44 mg/kg ketamine (Woerden, Netherlands) and 10 mg/kg xylazine (Alfazyne, Woerden, Netherlands), the area between the shoulders on the back was shaved and disinfected to isolate and culture the ad-MSCs. A 5-cm incision was made on the skin and 4 g of subcutaneous adipose tissue were collected and the area was sutured and post-surgical care was undertaken. To remove the blood cells, the tissues were washed three times with phosphate buffered saline (PBS; Gibco, USA) containing 1% penicillin and streptomycin (Gibco, USA). 

The tissue samples were minced in small pieces and digested in 0.2% collagenase type II (Gibco, USA) at 37 °C on a shaker for 40 min. The resultant mixture was filtered and centrifuged (5 min, 1500 ×g) and the pellet was re-suspended in 5 mL Dulbecco’s modified Eagles medium (DMEM; Gibco, USA). The suspension were transferred into 88% DMEM supplemented with 10% fetal bovine serum (FBS; Gibco, USA), and 1% penicillin and streptomycin, and were cultured at 37 °C in an incubator with 5% CO_2_ and saturated humidity. After 72 h, the medium was removed and the culture plate was washed with PBS and a new DMEM culture media supplemented with 10% fetal bovine serum, and 2 mM L-glutamine (Invitrogen, Netherlands) and 1% penicillin and streptomycin was added and transferred into CO_2_ incubator at 37 °C, 5% CO_2_ and saturated humidity. 

When cells were confluent, they were detached with 0.5 mM EDTA/0.05% trypsin (Gibco, USA) for 5 min at 37 °C and were later replated. So a homogeneous population of Ad-MSCs was obtained. Cultured MSCs were evaluated morphologically and by RT-PCR for expression of mesenchymal markers. 

The MSCs used for implantation were at passage 3. In the third passage, the cells at the logarithmic growth phase were collected and enumerated using a hemocytometer, and resuspended in frozen solution including 10% dimethyl sulfoxide (DMSO; MP. USA) and 90% FBS. The cell suspension was aliquot into sterile plastic cryovials labeled with the passage number, freezing serial number, and the date. The vials were sealed and kept at -20 °C for 60 min and then were transferred to -70 °C for 24 h, and ultimately into liquid nitrogen for long-term storage.

Before surgical treatment, the cryovials were removed from the liquid nitrogen and quickly thawed in a 37 °C water bath. When the ice clump was almost thawed, 1 mL of cell culture medium (88% DMEM, 10% FBS, and 1% penicillin and streptomycin) was added, centrifuged at 1500 rpm and the cells were transferred into a flask with gently blown into uniform single cell suspension, and transferred into CO_2_ incubator at 37 °C, 5% CO_2_ and saturated humidity. 

Twelve rabbits were divided equally into two groups including stem cell-treated and control groups. In cell-treated group, 2×10^6^ cells suspended in 1 mL of DMEM medium was aseptically injected into articular cartilage full-thickness defect. The control group received 1 mL of DMEM without any cells. Post-surgical care was undertaken by administration of 1.1 mg/kg flunixin meglumine (daily, 3 days, Flunex, Razak, Iran) and 50,000 IU/kg penicillin (daily, 5 days, Razak, Iran). Rabbits were euthanized by intravenous injection of a lethal dose of sodium thiopental (Nesdonal, France). All rabbits were restricted in their cage for any activity and mobilization till 4 and 8 weeks when euthanized. 

For histological evaluation, 4 and 8 weeks after the allotransplantation of the Ad-MSCs, in both cell-treated and control groups, three rabbits were sacrificed 4 weeks after treatment measures and three rabbits identically after 8 weeks. To reach the knee joint, the animals were sacrificed and placed on an operating table, shaved around the knee joint area, and arthrotomy was undertaken similarly as during transplantation to re-inspect the intra-articular structures. The degree of cartilage repair was grossly assessed for any possibilities of rejection or infection, such as presence of severe inflammation or extensive fibrosis, discoloration, irregularity, presence of any depression or bulging of repaired tissues in the defect area and state of the border with adjacent normal cartilage tissues. 

Full-thickness samples that were provided from each group at 4 and 8 weeks post-implantation were fixed in 10% formaldehyde, decalcified in 10% nitric acid for three days, dehydrated in graded ethanol, and embedded in paraffin wax. Then 5-µm paraffin-embedded sections were provided and deparaffinized. Each specimen was stained with hematoxylin and eosin (H&E), 0.1% safranin-O solution (Sigma, USA), and with toluidine blue (Sigma, USA). Each specimen was graded semi-quantitatively upon nature of the predominant tissue, regularity of the surface, thickness of the repair, matrix staining, structural integrity, apposition between the repaired cartilage and surrounding normal cartilage, freedom from degenerative signs in repair tissue and from degenerative changes of the surrounding normal cartilage as described before (Table 1) [16,17].

**Table 1:** Histological grading scale for evaluation of post-surgical articular regeneration [17].

Features	Scores
*Nature of the predominant tissue*	
Cellular morphology	4: Hyaline articular cartilage 2: Incompletely differentiated mesenchyme 0: Fibrous tissue or bone
Safranin-O staining of the matrix	3: Normal or nearly normal 2: Moderate 1: Slight 0: None
*Structural characteristics*	
Surface regularity	3: Smooth and intact 2: Superficial horizontal lamination 1: Fissure—25% to 100% of the thickness 0: Severe disruption, including fibrillation
Structural integrity	2: Normal 1: Slight disruption, including cysts 0: Severe disintegration
Thickness	2: 100% of normal adjacent cartilage 1: 50% to 100% of normal cartilage 0: 0% to 50% of normal cartilage
Bonding to the adjacent cartilage	2: Bonded at both ends of graft 1: Bonded at one end, or partially at both ends 0: Not bonded
*Freedom from cellular changes of degeneration*	
Hypocellularity	3: Normal 2: Slight 1: Moderate 0: Severe
Chondrocyte clustering	2: No clusters 1: <25% of the cells 0: 25% to 100% of the cells
Freedom from degenerative changes inadjacent cartilage	3: Normal cellularity, no clusters, normal staining 2: Normal cellularity, mild clusters, moderate staining 1: Mild or moderate hypocellularity, slight staining 0: Severe hypocellularity, poor or no staining

SPSS^®^ for Windows^®^ ver 11.5 (SPSS, Chicago, IL, USA) was used for statistical analysis. Mann-Whitney U test was used to compare the histotological criteria between groups. A p value <0.05 was considered statistically significant.

## RESULTS

**Figure 1:**
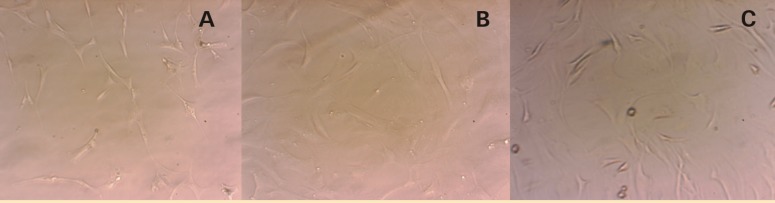
From left to right; 1^st^ (A), 2^nd^ (B) and 3^rd^ passage (C) of Ad-MSCs of rabbit (×40).

**Figure 2:**
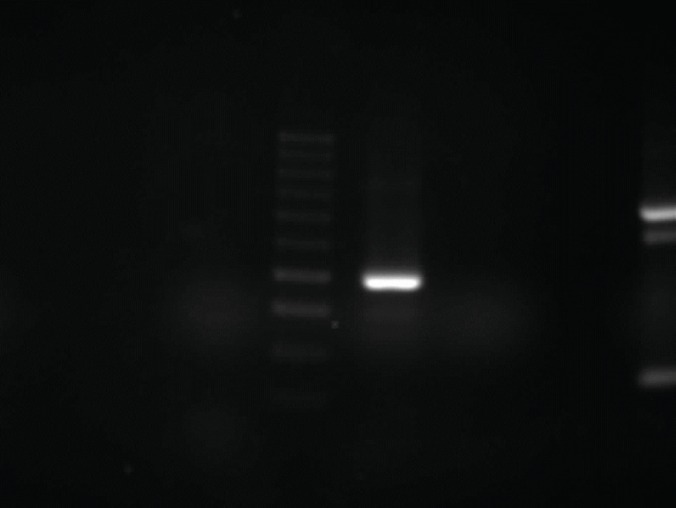
Positive expression of CD73 and negative expression of CD45 shown by RT-PCR.

Fibroblastic adherent cells were seen in the culture flask and in all subsequent subcultures (Fig 1). It was noticed that all cells were positive for expression of CD73 and negative for CD45 by RT-PCR (Fig 2). At 4 and 8 weeks post-transplantation, no rejection, infection, presence of severe inflammation, fibrosis, discoloration, irregularity, depression or bulging of repaired tissues were seen. Neo-formed tissues in transplanted cell group were translucent with a smooth and intact surface and less irregularity. In the control group receiving no cells, a depression in the defect area was visible. The articular surfaces in the defect site of transplanted cell group were relatively smoother than in the control group and their coloration was closer to that of surrounding normal cartilage in comparison to the control knees. Furthermore, in transplanted cell group; the border areas of defects were less distinct and depressions were less obvious than the control knees. 

**Figure 3:**
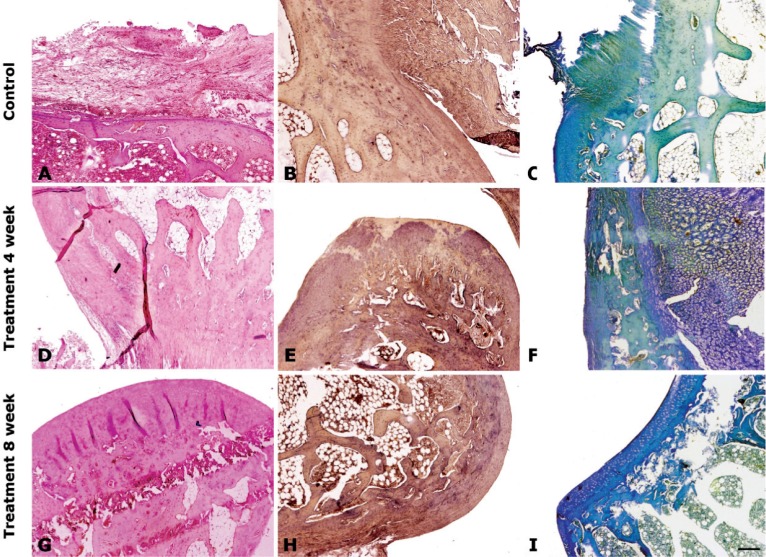
Histopathological evaluations of post-surgical articular regeneration after the allotransplantation of the Ad-MSCs onto the knee chondral defect in rabbits. H&E staining visualized the gross morphology (left panel); safranin-O staining assessed proteoglycan content (middle panel); and toluidine blue staining assessed glycosaminoglycans formation (right panel). Control group, after treatment with cell culture media without cell. Treatment groups, 4 and 8 weeks after treatment with Ad-MSCs. A) Cartilage fibrillation

**Figure 4:**
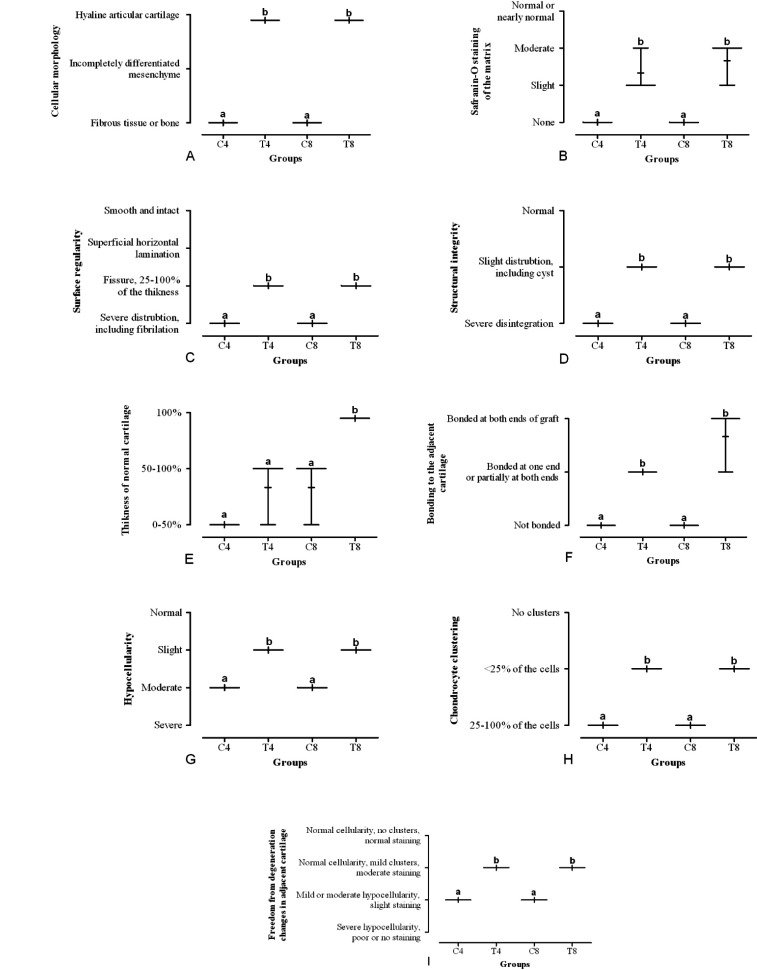
Box and whisker plots of histological and histochemical grading scale for evaluation of post-surgical articular regeneration [84] after the allotransplantation of the Ad-MSCs onto the knee chondral defect in rabbits. The same lower-case letters indicate a significant (p<0.05, Mann-Whitney U test) difference between comparable groups. C4 and C8 represent control groups 4 and 8 weeks, respectively after treatment with cell culture media without cell. T4 and T8 represent rabbits 4 and 8 weeks, respectively after treatment with Ad-MSCs.

When specimens were evaluated histologically, in transplanted cell group the knees were studied for structural characteristics, the nature of the predominant tissue, being free from inflammation, inflammatory cells and degenerative changes in adjacent cartilage in comparison to the control knees (Fig 3). After 8 weeks in post-transplantation group, the experimental knees were superior in overall score when compared to other groups (Fig 4).

Among animals after 4 and 8 weeks post-transplantation, experimental knees were smooth and had intact surface with hyaline articular cartilage and good column alignment of chondrocytes without cell clustering identical to the morphology of native cartilage (Figs 3 and 4). In the repair tissue, a vigorous repair process occurred between the repair and the adjacent tissues while in the control knees, a significant gap and reduced safranin-O positive staining was noticed in the border area stretching to subchondral bone. Moreover, the size of chondrocyte was relatively smaller than normal in the mid-zone. 

After 4 weeks of cell transplantation in the experimental knees, articular contours were restored, but the histological repair process appeared slower, more irregular and more immature than in groups after 8 weeks of the transplantation. The control knees showed a poor healing process with hypocellularity and cell number showed a decreasing trend in the defect area (Figs 3 and 4). Inflammatory responses, such as cysts, were seen in none of images.

In histological scorings, the repaired tissue in the experimental knee was overall superior to that in the control knee, which showed the adding value of transplanted cells (Fig 4). This overall higher score was more prominent after 8 weeks of cell transplantation in the experimental knees denoting to an overall higher cartilage repair of all parameters regarding the nature of the predominant tissue, structural characteristics, freedom from cellular changes of degeneration and freedom from degenerative changes (Fig 4).

## DISCUSSION

In this study, safety, feasibility and regenerative potential of Ad-MSCs in knee articular cartilage full-thickness defects in rabbit were assessed. No adverse effects were seen in Ad-MSCs transplantation group. Tissue repair was visible after 4 weeks based on histological findings and after 8 weeks for knee articular cartilage full-thickness defects. The results were much more prominent when compared to the control group. This difference may be due to less time for implanted cells in 4 weeks group when compared to the 8 weeks group [18]. 

Cell-based therapy in the form of autologous chondrocyte implantation (ACI) was first described in 1994 [19] followed by several researchers to increase the repair potential of damaged cartilage [6]. This technique has difficulty in obtaining an adequate number of chondrocytes, and also requires a formal arthrotomy. Some authors even reported that ACI results at 2 and 5 years were not different from simpler microfracture technique [20].

The safety and efficacy of cartilage repair in the human knee using arthroscopic microfracture and injections of MSCs and hyaluronic acid were previously reported [2]. In cell-based therapy, the use of MSCs for cartilage repair in humans as an alternative to chondrocytes has also gained some momentum [21]. 

Some authors reported augmenting the scaffolds with either autologous articular chondrocytes cultured from biopsies of non-weight-bearing local cartilage or MSCs that lead to better regeneration of the cartilage and subchondral bone. The MSCs may themselves differentiate into the desired phenotype and can recruit and activate local MSCs toward regeneration, or may reactivate local differentiated cells for this purpose. 

So the early regenerative effect in cell-treated group may be because of paracrine or trophic effects of MSCs on residing cells rather than their innate differentiation potential. This paracrine effect of MSCs was reported by others to be more important than the differentiation potential of the cells [22] as it was shown that only 8%–33% of the cell population in the defect arose from the implant itself [23] and in the case of implanting chondrocytes, they had no additional contribution at all [24]. 

Wakitani, *et al* [25], compared 2 groups of patients who had undergone high tibial osteotomy. The first group received implantation of collagen gel scaffold embedded with bone marrow-derived stem cells while the second group received cell free scaffolds implantation. These authors managed to show better arthroscopic and histologic scores in the MSCs-treated group [25]. The clinical outcomes of patients treated with first generation ACI to patients treated with bone marrow-derived MSCs were previously compared. The latter was demonstrated to have a better proliferation rate than chondrocytes and had the capacity to differentiate to various tissues, such as bone and cartilage [26]. 

Viable injected cells were recovered in a goat knee with induced arthritis [27]. In porcine, *in vivo* tracing of green fluorescent protein (GFP) labeled MSCs showed that these cells were localized at the site of a surgically created full-thickness chondral defect and resulted into formation of neo-cartilage [28]. In porcine, it was shown that intra-articular injection of MSCs and hyaluronic acid (HA) was effective in healing of full-thickness femoral condyle cartilage defects while *in vivo* tracing of labeled cells confirmed the presence of injected MSCs in the neo-cartilage [28]. In goat model, MSCs were successfully used in treatment of osteochondral defects [29].

In rat after injection of GFP-labeled MSCs into the knees, it was found that the injected cells were mobilized to the injured sites [30]. Again in rat model, it was found that intra-articular injections of GFP and MSCs along with a bone marrow stimulation procedure were more effective in repair of a chronic osteochondral lesion indicating that the injected MSCs “home in” onto the site of injury. They hypothesized that the induced growth factors were attributed to the injected stem cells adhered to the site of injury, preventing them from escaping and finally contributed in differentiation to chondrocytes [31]. It seems that two temporarily distinct injury-related signals first induced MSCs to home in onto the site of injury and then a second local signal lead to differentiation of MSCs into the desired cell type to facilitate the repair of the injured tissue [32]. 

In our study, we isolated and evaluated the role of implantation of Ad-MSCs in repair of rabbit cartilage defects as a novel cell source. After 4 and 8 weeks, it was shown that the injured knees had histologically and morphologically, a superior cartilage repair in comparison to the control group. As articular cartilage is a highly differentiated, avascular tissue with a low self-regeneration capacity, many researchers tried to increase the repair potential of damaged cartilage using cell-based therapies, such as stem cell implantation similar to our study [12, 33, 34]. 

It was demonstrated that there might be possible interactions between human nucleus pulposus (NP) cells and MSCs mediated by secreted proteins [35]. It was shown that the stimulus from MSCs in the host could modulate the therapeutic activities of transplanted MSCs, which suggest that an interaction between MSCs and host cells plays an essential role in cartilage regeneration describing our findings [36, 37]. In addition, the anti-inflammatory and immune-modulating properties of MSCs may also be involved in the cartilage repair mechanisms, especially the anti-inflammatory effects affecting the intra-articular microenvironment revealing the efficacy of our treatment protocol in rabbits [38, 39].

There are similar studies on application of allogenic Ad-MSCs as an alternative option in cell therapy of osteochondral defects [33, 34]. Other sources were reported to be used in cartilage repair such as bone-marrow-derived stem cells with different outcomes [12]. Human umbilical cord blood MSCs were used in repair of articular cartilage defects as a safe source with *in vitro* chondrogenic differentiation potential. Even its *in vivo* cartilage regeneration potential is still under question [25, 40, 41]. 

Ad-MSCs were shown to be a safe and an available tissue source in surgical wards that could be collected in a non-invasive way with suitability for immediate transplantation *in vivo* and with hypo-immunogenic properties denoting to the important role of these cells in cell transplantation purposes while our findings confirmed their effect in healing of knee chondral defects of rabbit model [34]. Another study demonstrated the preclinical safety and feasibility of freshly isolated and cultured Ad-MSCs [42]. We also believe that the mechanism of cartilage regeneration in our model may be associated with chondrogenic differentiation, the paracrine action of Ad-MSCs, and their immunomodulatory effects. The cell concentration for the regeneration of the defect may also be another issue [43].

We showed that Ad-MSCs, as an available and non-invasive source in surgical wards, could be safely administered for transplantation purposes in knee osteochondral defects. A longer follow up of more than eight weeks is suggested to clarify the healing potential of these cells after this period.
